# First study of susceptibility and resistance status to pyrethroids insecticides in *Anopheles (Cellia) sergentii* (Theobald, 1907) from Southern Tunisia

**DOI:** 10.4314/ahs.v18i1.8

**Published:** 2018-03

**Authors:** Ahmed Tabbabi, Jabeur Daaboub

**Affiliations:** Department of Hygiene and Environmental Protection, Ministry of Public Health, Tunis, Tunisia

**Keywords:** *Anopheles sergentii*, Pyrethroids insecticides, Tunisia

## Abstract

**Background:**

Insecticide resistance is an important threat to malaria control. *Anopheles (An.) sergentii* proved to be the number one vector in the oases and may be of a particular interest in projection of the future trends of the disease in Tunisia.

**Objectives:**

Resistance status to pyrethroids insecticides in *An. sergentii* was evaluated for the first time in Tunisia.

**Methods:**

Diagnostic resistance tests to pyrethroids insecticides were conducted on late third and early fourth larvae of *An. sergentii* collected in Southern Tunisia.

**Results:**

The level of resistance to permethrin and deltamethrin varied from 1.9 to 5.77 and from 2.75 to 4.63, respectively. The highest resistance was recorded in sample # 3 to the two used insecticides. Synergists showed that esterases and glutathione-S-transferase were not involved in the resistance to any of the evaluated insecticides. In contrast, cytochrome-P450 monooxygenases played a role in the detoxification of two among three studied samples. Positive correlations between larval tolerance to both Permethrin/DDT and Deltamethrin/DDT were recorded indicated target site insensitivity.

**Conclusion:**

Continued monitoring of insecticide susceptibility and generating complementary data on mechanisms of resistance using molecular and biochemical methods is essential to ensure early detection of insecticide resistance in potential malaria vectors in Tunisia.

## Introduction

In Tunisia, malaria was the most important vector-born-disease until its elimination in 1980^1^. It is caused by parasites of the genus *Plasmodium (P.)* which are transmitted to humans via the bites of females mosquitoes of the genus *Anopheles*. Historically, only three species of *anophelines* are known to be the malaria vectors: *Anopheles (An.) labranchiae* in Northern Tunisia, and *An. Sergentii* and *An. multicolor* in Southern Tunisia (Wernsdorfer W and Iyengar MO, unpublished data). *An. sergentii* proved to be the number one vector in the oases and may be of a particular interest in projection of the future trends of the disease in Tunisia. Due to the climate change, Tunisia was at risk of lack of water and reduction in crop productivity that's why the underground water reserves in Southern Tunisia were used in irrigation/agricultural projects. Under these conditions, the malaria vector of oases (*An. sergentii*) may easily emerge and settle down. These observations could be associated with the increase of the annual incidence of imported cases of malaria and highlight the risk of a resumption of the disease transmission in Tunisia^2^–^5^.

It should be noted that since 1903 and mainly after the World War II, intensive chemical control of malaria vectors have led to successful interruption of autochthonous malaria transmission^3^,^6^. However, this has been limited by the development and spread of resistance^7^. The problem of insecticide resistance is very real and growing in Tunisia^7^–^9^. Effects of climate change and the concern that the mosquitoes are becoming resistant to the entire classes of insecticide in use including pyrethroids may aggravate the situation. It is important to note that resistance of *Anopheles* mosquitoes to pyrethroid insecticide have never been studied in Tunisia. Their susceptibility to organophosphates insecticides was only approximated by some bioassays on small sample without estimation of involved mechanisms^10^. Generally, two major mechanisms are involved in pyrethroids insecticides resistance: (1) the knockdown resistance phenotype, (kdr) occurs due to a point mutation in the voltage gated sodium channel in the central nervous system, the common target of pyrethroids and DDT; and (2) the increased metabolic detoxification of insecticides including three major enzyme superfamilies: Esterases, Multi function Oxidases P450 and Glutathion-S Transferases^11^,^12^.

The bionomics of *An. Sergentii*, its ability to transmit strains of tropical *P. falciparum* and its susceptibility status to insecticides are unknown and poorly documented. It is the specific characteristics that need to be studied in order to improve epidemiological surveillance. The present study aimed to evaluate the susceptibility and resistance status to pyrethroids insecticides in *An. sergentii* (Theobald, 1907) for the first time in Tunisia.

## Material and methods

### Study area and mosquito collection sites

**Figure 1:**
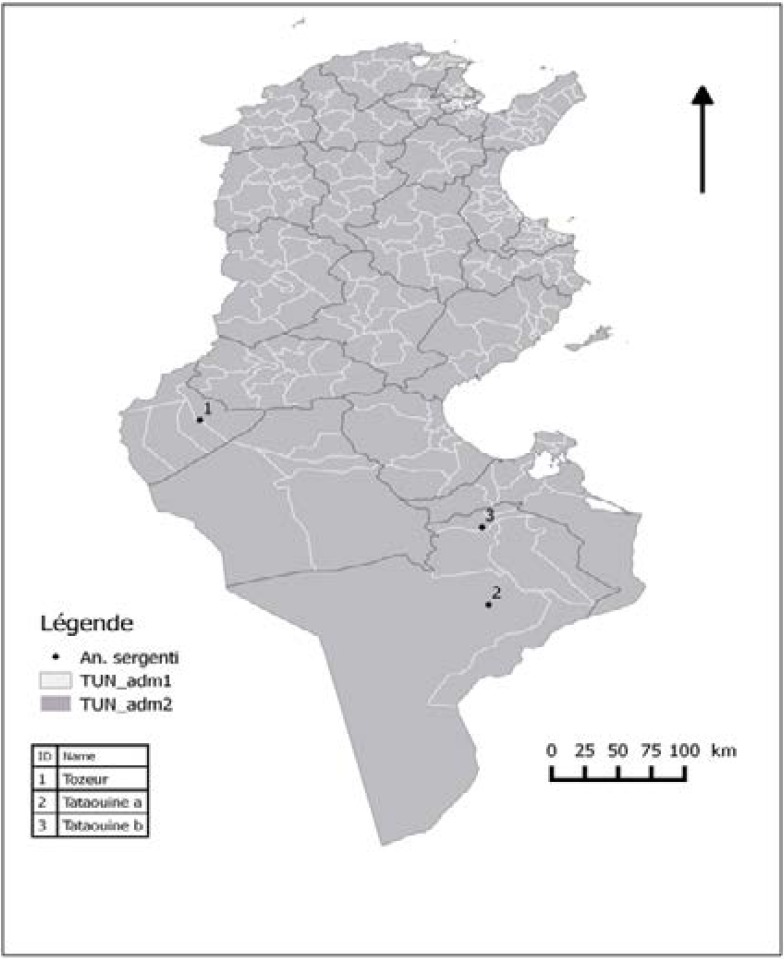
Geographic origin of Tunisian populations

The study was carried out on *An. sergentii* mosquitoes from three breeding sites in SouthEast and SouthWest Tunisia (Figure 1) between September and November 2016. Mosquitoes were collected from a ditch, river and water pond. The studied areas are not submitted to agricultural pest control but frequently to mosquito control using organophosphates and pyrethroids insecticides (Table 1).

**Table 1:** Geographic origin of Tunisian populations of *An. (Cellia) sergentii* Theobald, 1907, breeding site characteristics, and insecticide control

Code	Governorate	Breeding sites	Date of collection	Mosquito control (used insecticides)	Agricultural pest control
1	Tozeur	Ditch	Sep. 2016	Frequent (C, Pm, F, P, D)	None
2	Tataouine a	River	Nov. 2016	Frequent (C, Pm, P, D)	None
3	Tataouine b	Water pond	Nov. 2016	Frequent (C, Pm, P, D)	None

C : Chlorpyrifos; Pm : Pirimiphos methyl; F : Fenitrithion; P : Permethrin; D : Deltamethrin

### Mosquito strains

Late third and early fourth larvae of *An. sergentii* were collected and used for insecticide and synergist bioassays. The morphological identification was carried out using identification software of mosquitoes of the Mediterranean Africa^13^. A sensitive strain collected from Southern Tunisia and showed high susceptibility to pyrethroid insecticides was used for comparison with resistant collected populations.

### Insecticides and synergists

Three technical-grade insecticides were used for bioassays: the pyrethroid permethrin (94.6Vo, ICI Americas, Inc., Richmond, CA), the pyrethroid deltamethrin (95.7Vo, ICI Americas, Inc., Richmond, CA), and the organochloric DDT (99.9Vo; Mobay). Two synergists were used to help detect detoxification enzymes involved in resistance: S,S,Sributyl phosphorothioate (DEF), an esterase inhibitor, and piperonyl butoxide (PB), an inhibitor of mixed function oxidases.

### Diagnostic resistance and synergy tests

Diagnostic tests were conducted on late third and early fourth larvae of *An. sergentii* using the standard methods of Raymond et al^14^. Three replicates of 20 mosquitoes were tested against each concentration. Twenty mosquito larvae were placed in 100 ml cup containing 99 ml of distilled water. A series of test concentrations, dissolved in ethanol, were added to give the aimed final concentrations. Five replicates without insecticides were used for control. A series of concentration was used to obtain mortality response from 0 to 100%. To verify that the concentrations of synergist were below toxic levels, standard sub lethal doses of 0.08 mg/l for DEF, and 2.5 mg/l for Pb, 4 hours before the addition of the insecticide were used.

### Data analysis

Mortality data were analyzed using log dose-probit mortality software developed by Raymond et al^15^ based on Finney^16^. This program is able to do the probit regression analysis, the comparison of probit lines by testing parallelism of slopes, and to produce confidence limits of resistance ratios. When mortality data were not linear but rather displayed a plateau, values for lethal concentrations were approximated graphically after plotting on log-probit paper. Frequencies of resistant individuals were compared using chi-squared contingency tables.

## Results

As shown in Table 2, the results on bioassays tests showed that *An. sergentii* was resistant to permethrin and deltamethrin but not exceeded 5-folds. The level of resistance varied from 1.9 to 5.77 and from 2.75 to 4.63, respectively. The highest resistance was recorded in sample # 3 to the two used insecticides. Slope values reported the linearity of all studied samples (p>0.05) indicating a homogeneity of considered phenotype in different studied strains (Table 2).

**Table 2a:** Permethrin resistance characteristics of Tunisian *An. (Cellia) sergentii* Theobald, 1907 in presence and absence of synergists DEF and Pb

Population	Permethrin			Permethrin +DEF						Permethrin +Pb					
	
	LC_50_ in µg/l (a)	Slope ± SE	RR_50_ (a)	LC_50_ in µg/l (a)	Slope ± SE	RR_50_ (a)	SR_50_ (a)	RSR	LC_50_ in µg/l (a)	Slope ± SE	RR_50_ (a)	SR_50_ (a)	RSR
**Sensitive strain**	2.2 (1.5–4.6)	2.12 ± 0.74	-	1.8 (1.1–2.5)	2.3 ± 0.45	-	1.22 (0.9–2.7)	-	1.4 (0.89–2.4)	2.12 ± 0.87	-	1.57 (0.94–3.2)	-
**1-Tozeur**	4.2 (3.4–5.9)	1.47 ± 0.13	1.9 (1.1–3.1)	5.3 (3.9 – 6.3)	1.13 ± 0.85	2.94 (1.6–3.8)	0.79 (0.14–1.22)	0.64	5.4 (4.9–6.4)	1.65* ± 0.3	3.87 (2.2–5.6)	0.77 (0.24–0.97)	0.49
**2-Tataouine a**	6.4 (5.2–7.8)	3.12 ± 0.77	2.9 (1.78–4.25)	4.8 (3.2–6.4)	1.92 ± 0.21	2.66 (1.4–3.89)	1.33 (0.54–1.99)	1.09	4.98 (3.3–6.45)	2.97 ± 0.27	3.55 (2.45–6.5)	1.28 (1.1–1.78)	0.81
**3-Tataouine b**	12.7 (10.9–15.3)	2.22 ± 0.32	5.77 (3.45–7.29)	10.89 (8.52–12.78)	1.45 ± 0.28	6.05 (3.7–8.21)	1.16 (0.8–2.6)	0.95	10.8 (8.8–12.4)	1.26 ± 0.34	7.71 (4.7–8.9)	1.17 (0.8–2.5)	0.74

**Table 2b:** Deltamethrin resistance characteristics of Tunisian *An. (Cellia) sergentii* Theobald, 1907 in presence and absence of synergists DEF and Pb

Population	Deltamethrin			Deltamethrin +DEF						Deltamethrin +Pb					
	
	LC_50_ in µg/l (a)	Slope ± SE	RR_50_ (a)	LC_50_ in µg/l (a)	Slope ± SE	RR_50_ (a)	SR_50_ (a)	RSR	LC_50_ in µg/l (a)	Slope ± SE	RR_50_ (a)	SR_50_ (a)	RSR
**Sensitive** **strain**	1.9 (1.2–2.8)	2.87 ± 0.76	-	1.7 (1.0–2.8)	1.99 ± 0.12	-	1.11 (0.9–2.1)	-	1.3 (0.77–1.86)	2.1 ± 0.45	-	1.46 (0.85–2.22)	-
**1-Tozeur**	7.5 (5.8–8.9)	1.29 ± 0.45	3.94 (2.1–5.7)	9.9 (8.4–11.8)	1.45 ± 0.35	5.82 (4.6–7.42)	0.75 (0.35–1.5)	0.67	1.2 (0.52–1.65)	1.23 ± 0.41	0.92 (0.47–1.45)	6.25 (4.56–7.81)	4.28
**2-Tataouine a**	4.9 (2.6–6.7)	1.75 ± 0.22	2.57 (1.18–3.45)	7.48 (5.78–9.45)	0.89 ± 0.15	4.4 (3.5–7.9)	0.65 (0.22–1.3)	0.58	3.5 (2.4–4.9)	0.81* ± 0.24	2.69 (1.79–3.34)	1.4 (0.87–1.97)	0.95
**3-Tataouine b**	8.8 (7.2–9.7)	1.53 ± 0.16	4.63 (3.2–6.7)	10.2 (8.2–12.8)	1.41 ± 0.65	6 (4.4–8.45)	0.86 (0.5–1.7)	0.77	0.85 (0.24–1.56)	1.22 ± 0.53	0.65 (0.23–1.42)	10.35 (9.33–12.54)	7.12

(a), 95% CI;

* The log dose-probit mortality responses is parallel to that of S-Lab. RR50, resistance ratio at LC_50_ (RR_50_=LC_50_ of the population considered / LC_50_ of Slab); SR_50_, synergism ratio (LC_50_ observed in absence of synergist / LC_50_ observed in presence of synergist). RR and SR considered significant (P<0.05) if their 95%CI did not include the value 1. RSR, relative synergism ratio (RR for insecticide alone / RR for insecticide plus synergist).

In the presence of Pb (2.5 mg/l applied four hours before the treatment), the toxicity of deltamethrin significantly increased in samples # 1 and 3 (Table 2). The median-lethal doses of deltamethrin (7.5 and 8.8 respectively) were about 7 and 10 times lower than that obtained without synergists. This indicates that cytochrome-P450 monooxygenases played a role in the detoxification of these two samples.

Applying DEF at 0.08 mg/l four hours prior to treatment with permethrin and deltamethrin had little effect on toxicity of both compounds (Table 2). The median-lethal dose of the two used compounds was almost unchanged. According to the results obtained from the use of S.S.S. phosphotrithiate trybutil synergist, it was shown that esterases and glutathione-S-transferase were not involved in the resistance to any of the evaluated insecticides.

A positive correlations between larval tolerance to both Permethrin/DDT and Deltamethrin/DDT were recorded (Spearman rank correlation, (r) = 0.83 and 0.91, respectively (P<0.01)) indicated target site insensitivity (Voltage-Gated Sodium Channel).

## Discussion

The results of susceptibility tests using standard methods of Raymond et al^14^, showed that *An. sergentii* was lowly resistant to permethrin and deltamethrin insecticides. The difference in resistance levels from one population to another can be explained by their behavioural resistance mechanism. It should be noted that resistance of *An. sergentii* has never been reported to pyrethroids in Tunisia. Preliminary tests on small sample showed their susceptibilities to organophosphates insecticides in central Tunisia and Morocco^10^,^17^. Note that various levels of resistance to organophosphate and pyrethroids were also reported in the secondary malaria vector *An. nuneztovari*^18^. The low and moderate resistance in *An. sergentii* to organophosphates and pyrethroids insecticides presents greater opportunity for managing resistance in Tunisia. Several authors showed the positive correlation between pyrethroids resistance mechanisms and selection pressure caused by DDT insecticide used for malaria vector control between 1964 and 1978 in Tunisia^7^ without forgetting the recent frequent mosquito control using organophosphates and pyrethroids insecticides. Therefore, periodic monitoring the resistance status, its mechanisms and study on cross-resistance are necessary to evaluate the insecticides and solve problem of control programs. Noting that other environmental factors like the pollution and the biotic interactions between vectors and other organisms may affect mosquito responses to pyrethrois insecticides.

Several standard methods are frequently used to evaluate resistance to insecticide in malaria vectors. Bioassays are the mains approaches to estimate cross-resistance to commonly used classes of insecticides by identification of resistance mechanisms^19^. In this context, cross-resistance to both Permethrin/DDT and Deltamethrin/DDT were tested and showed a positive correlation indicating indicated target site insensitivity as common mechanism (Voltage-Gated Sodium Channel). These results confirm the above findings and the selection pressure with DDT during the national malaria eradication program in the 60s and 70s. Many genetic, biological and operational parameters^20^–^22^ oriented the evolution of insecticides resistance which is very complex. We cited the biological parameters associated with the life cycle of the malaria vector such as the number of generation and the rate of reproduction, migration, and isolation, the genetic parameters including polygenic resistance, dominance, fitness cost and gene interaction. Operational parameters that include the method and frequency of application, dosage and residual activity of the insecticides as well as insecticide coverage.

The metabolic resistance was investigated using synergists which act by blocking metabolic pathways that would otherwise break down insecticides, then restore the susceptibility to the insecticide^23^–^25^. Synergist's bioassays give us preliminary information on detoxification enzymes co-involved with kdr mutations in *An. sergentii* resistance in Tunisia. Our findings showed that esterases and glutathione-S-transferase were not involved in the resistance to any of the evaluated insecticides. In contrast, cytochrome-P450 monooxygenases played a role in the detoxification of two among three studied samples and partial effect for kdr mutation in shaping resistance to DDT, permethrin and deltamehtrin in field-collected populations^26^,^27^. Indeed, several studies showed the major contribution of target site in the recorded resistance^28^. On the other hand, Raymond et al^29^ reported the additive action of detoxification enzymes and target site. DDT, permethrin and deltamethrin resistance was reported in field populations of *An. albimanus* from Guatemala, whereas full susceptibility was recorded in *Anopheles* mosquitoes from El Salvador and Belize^30^,^31^. The strains from Guatemala showed significant increase in activities of esterase and/or oxidase as measured by spectrophotometer showing their involvement in pyrethroid-resistance^30^.

## Conclusion

The low pyrethroids resistance observed in Tunisia malaria vector is particularly interesting, because it leaves a range of tools useable by vector control services. Continued monitoring of insecticide susceptibility and generating complementary data on mechanisms of resistance using molecular and biochemical methods is essential to ensure early detection of insecticide resistance in potential malaria vectors in the region.
